# Preliminary *In Vitro* Evaluation of Genistein Chemopreventive Capacity as a Result of Esterification and Cyclodextrin Encapsulation

**DOI:** 10.1155/2015/262930

**Published:** 2015-05-26

**Authors:** Corina Danciu, Codruta Soica, Cristina Dehelean, Istvan Zupko, Erzsebet Csanyi, Iulia Pinzaru

**Affiliations:** ^1^Department of Pharmacognosy, University of Medicine and Pharmacy “Victor Babes”, Eftimie Murgu Square, No. 2, 300041 Timisoara, Romania; ^2^Department of Pharmaceutical Chemistry, University of Medicine and Pharmacy “Victor Babes”, Eftimie Murgu Square, No. 2, 300041 Timisoara, Romania; ^3^Department of Toxicology, University of Medicine and Pharmacy “Victor Babes”, Eftimie Murgu Square, No. 2, 300041 Timisoara, Romania; ^4^Department of Pharmacodynamics and Biopharmacy, University of Szeged, Eotvos utca 6, Szeged 6720, Hungary; ^5^Department of Pharmaceutical Technology, University of Szeged, Dómtér 8., Szeged 6720, Hungary

## Abstract

The present study focuses on the synthesis and analysis of a genistein ester derivative with myristic acid followed by beta cyclodextrin encapsulation; physicochemical analysis using consecrated techniques such as FTIR, MS, DSC, and SEM revealed both a successful esterification and inclusion inside the cyclodextrin cavity. Cytotoxic effects were measured* in vitro* on three human cell lines: HeLa (cervix adenocarcinoma), A2780 (ovary carcinoma), and A431 (skin epidermoid carcinoma). The* in vitro* biological analysis exhibited rather poor antiproliferative results on all three tested cancer cell lines, behavior that may be due to the high stability of the complex within the* in vitro* environment.

## 1. Introduction

Complementary and alternative medicine offer opportunities and challenges for a different approach to a wide range of pathologies. Additionally, numerous vegetal products provided natural compounds that served as models for synthetic or semisynthetic molecules used in allopathic medicine. Also, in the twenty-one century, plants and plant materials deliver a vast number of structural templates for drug development [1–4].

The isoflavone genistein is one of the most intensely studied phytocompounds in the class of flavonoids. It is a secondary plant metabolite, characterized by pleiotropic biological activity [5–8]. Among its properties, a special interest has focused on its antiosteoporotic, cardioprotective, and chemopreventive effects. Asian epidemiological studies show that the consummation of soy foods containing isoflavones is directly correlated with a wide range of health benefits, including reduced incidence of breast and prostate cancers [9, 10]. In line with the currently discussed topic, genistein has shown* in vitro* antiproliferative activity on human breast cancer cells, human prostate cancer cells, human colon cancer cells, human gastrointestinal cancer cells, human renal cell carcinoma, and human melanoma [6, 11–16]. Furthermore, in a previous* in vivo* study we have shown that genistein possesses significant effects on tumour development in terms of tumour size, metastasis potential, and melanisation [6].

Numerous studies reported stronger antiproliferative activity for genistein derivatives compared to the parent compound [17–19]. It has been previously revealed that genistein esterification with various fatty acids may increase its biological activity both* in vitro* and* in vivo,* by raising its cellular uptake [20]. Furthermore it has been proven that esterification may improve genistein stability and plasmatic life [21]. Another possibility to improve genistein solubility and, by consequence, bioavailability involves its entrapment inside branched cyclodextrins [7, 22]. CDs are a class of oligosaccharides with toroidal structures having a hydrophilic exterior and hydrophobic interior, thus the capacity to form inclusion complexes with a large number of hydrophobic drugs whose water solubility will be significantly increased [23, 24].

The aim of this preliminary* in vitro* study is to assess the influence of genistein esterification on its antiproliferative activity on the selected cell lines; furthermore the incorporation of the newly synthesized ester inside beta-cyclodextrin was conducted in order to evaluate the potential of increased biological activity.

## 2. Materials and Methods

### 2.1. Reagents

Genistein (GEN) was purchased from Extrasynthese (France, purity > 95%), beta-cyclodextrin (BCD), from Cyclolab, Hungary, myristoyl chloride from Sigma-Aldrich, triethylamine from Merck, and all the other chemicals including the solvents (chloroform, ethyl acetate, methanol, and acetonitrile) were at least of analytical grade and used as received. All the solvent mixtures were defined as v/v. All substances were used as received.

### 2.2. Chemical Esterification of Genistein

**Figure 1:**
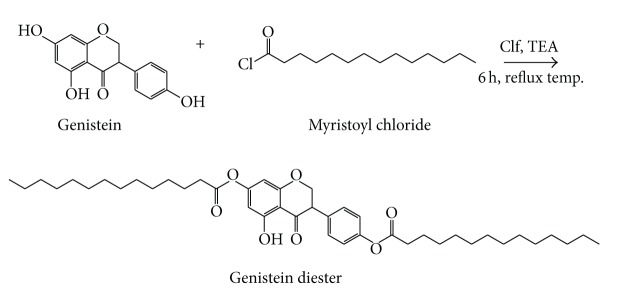
Chemical esterification of genistein using myristoyl chloride, in chloroform (Clf) media, in the presence of triethylamine (TEA), for 6 hours at reflux temperature. The solvent was removed by evaporation and the products were further purified by column chromatography thus achieving a highly pure genistein ester (GMAE) in 34–39% yield.

Genistein (1 mmol) was solubilized in 10 mL chloroform under magnetic stirring at 50°C. The temperature was adjusted at reflux temperature and triethylamine was slowly added to the solution. The esterification started after the dropwise addition of myristoyl chloride (2 mmol) in the solution. After 6 hours, the mixture was cooled down at room temperature, washed with water until neutral pH, and dried over anhydrous MgSO_4_. In order to obtain the crude precipitate, the solvent was removed by evaporation using a rotary evaporator; the products were further purified by column chromatography thus achieving a highly pure genistein ester (GMAE) in 34–39% yield. The schematic representation of genistein esterification is depicted in Figure 1.

### 2.3. Complexes Preparation

The CD and the active compounds were kneaded with 50% (v/v) water : ethanol solution until the bulk of solvent evaporated and a paste-type product was formed; the mixture was then dried at room temperature for 24 hours and put in the oven, at 105°C, for several hours until reaching constant weight. The final product was pulverized and sieved. All the binary products were prepared using 1 : 2 molar ratio drug : CD; the high content of CD was chosen in order to provide a higher water solubility as well as inclusion degree of the guest molecules.

### 2.4. TLC Analysis

The chemical synthesis reactions were qualitatively monitored through thin layer chromatography (silica gel plates 60 F254, Merck) using chloroform/ethyl acetate (6/4) as eluent. Both genistein and its esters were visualized under ultraviolet light (254 nm).

### 2.5. HPLC Analysis

Qualitative analysis of the samples was performed using a YL 9100 HPLC System, equipped with vacuum degasser (YL 9101), quaternary pump (YL 9110), column compartment (YL 9131), and spectrophotometric detector (YL 9120); the working conditions were Nucleosil 100 C-18, 250 × 4.6 mm × mm column, particle diameter 5 *μ*m, and wavelength 254 nm. Separation of various components from the reaction medium was carried out using water (0.1% acetic acid)/acetonitrile (0.1% acetic acid) as mobile phase with a flow rate of 1 mL/min, at 25°C.

### 2.6. FTIR Spectroscopy

FTIR spectra of genistein and genistein derivatives were recorded on the Perkin Elmer SPECTRUM 100 spectrometer using the UATR technique on 4000–400 cm^−1^ spectral range.

### 2.7. MS Spectroscopy

For NanoMate HCT MS experiments, the solution of genistein and its derivatives was prepared by dissolving the dry sample in pure methanol up to 10 pmol *μ*L^−1^. Mass spectrometry was conducted on a High Capacity Ion Trap Ultra (HCT Ultra, PTM discovery) mass spectrometer from Bruker Daltonics, Bremen, Germany. All mass spectra were acquired in the mass range (200–1500)* m/z*, with a scan speed of 8000* m/z* per second. The* m/z* scale of all mass spectra was externally calibrated using G2421A electrospray “tuning mix” from Agilent Technologies (Santa Rosa, CA, USA) as calibration standard. Following calibration procedure, the obtained mass accuracy was situated within the normal range of a HCT MS instrument.

All mass spectra were processed by Data Analysis 3.4. Software from Bruker Daltonics (Bremen, Germany), which allows signal extraction, smoothing, and subtraction. Proposals for molecular ion composition were made by exact mass calculation.

### 2.8. Differential Scanning Calorimetry (DSC)

The DSC measurements were conducted by using the Mettler Toledo DSC 821^e^ thermal analysis system with STAR^e^ thermal analysis program v. 9.1 (Mettler Inc., Schwerzenbach, Switzerland). Approximately 2–5 mg of genistein or its product was examined in the temperature range between 25°C and 350°C. The heating rate was 5°C min^−1^. Argon was used as carrier gas, at a flow rate of 10 L h^−1^ during the DSC investigation.

### 2.9. Scanning Electron Microscopy (SEM) Assay

The shape and surface characteristics of genistein and respective complexes were examined using a scanning electron microscope (Hitachi S4700, Hitachi Ltd., Japan). The samples were sputter coated with gold-palladium under an argon atmosphere using a gold sputter module in a high vacuum evaporator using SEM set at 15 kV.

### 2.10. MTT Proliferation Assay

Cytotoxic effects were measured* in vitro* on three human cell lines (ECACC; Salisbury, UK): HeLa (cervix adenocarcinoma), A2780 (ovary carcinoma), and A431 (skin epidermoid carcinoma). The cells were cultivated in minimal essential medium (Sigma-Aldrich, Budapest, Hungary) supplemented with 10% fetal bovine serum, 1% nonessential amino acids and an antibiotic-antimycotic mixture. Nearconfluent cells were plated into a 96-well plate at the density of 5000 cells/well and, after overnight standing, the medium containing the tested compound (at 10 or 30 *μ*M) was added. Following a 72-h incubation in a humidified atmosphere of 5% CO_2_ at 37°C the living cells were assayed by the addition of 20 *μ*L of 5 mg/mL MTT [3-(4,5-dimethylthiazol-2-yl)-2,5-diphenyltetrazolium bromide] solution. MTT was converted by intact mitochondrial reductase and precipitated as blue crystals during a 4-h contact period. The medium was then removed, the formazan crystals were solubilized in 100 *μ*L DMSO during a 60-min period of shaking at 25°C, and the absorbance was determined at 545 nm using a microplate reader. Wells with untreated cells were used as controls. All* in vitro* experiments were carried out on five parallel wells. Stock solutions of the tested substances (10 mM) were prepared with DMSO as solvent; the DMSO concentration (0.3%) of the medium did not have any significant effect on cell proliferation.

### 2.11. Statistics

One-Way ANOVA followed by Newman-Keuls posttest was used to determine the statistical difference between various experimental and control groups. Statistical analyses were performed with GraphPad Prism 4.0 (GraphPad Software, San Diego, CA, USA).

## 3. Results and Discussions

The structure of genistein myristate was elucidated using FTIR and MS spectra. The ester formation was confirmed by the IR spectral data; thus, the product showed signals corresponding to the band of the ester carbonyl group at 1755.14 cm^−1^ for GMAE, while the vibrations at 1703.06 cm^−1^ attributed to the carbonyl groups from fatty acids disappear in the derivatives' spectra. However, the specific carbonyl band exhibited by genistein at 1649.06 cm^−1^ can still be found in the derivatives' spectra. The mass spectrum of GMAE revealed the value* m/z* 688.98, which indicates the formation of genistein's diester.

**Figure 2:**
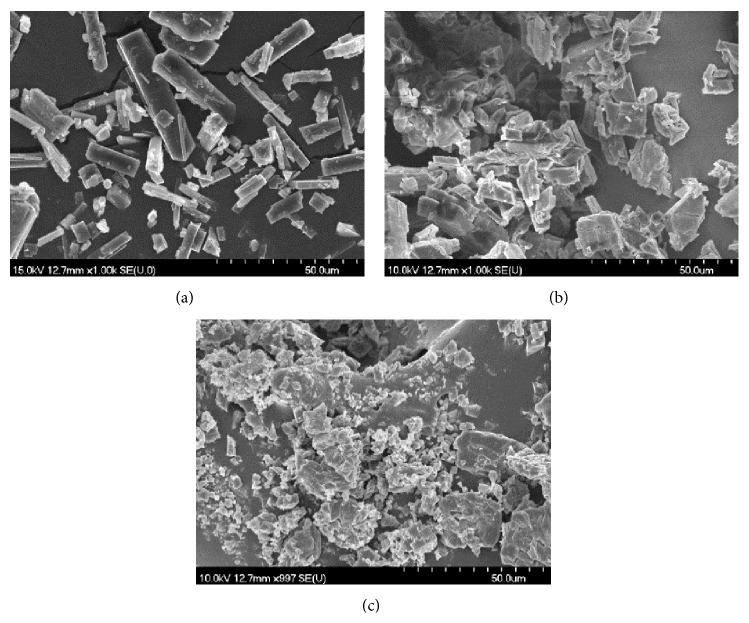
SEM pictures for (a) GEN, clear paralelipipedic crystals, (b) GMAE, irregular shaped crystals, and (c) GMAE-BCD, amorphous product indicating the formation of a distinct new product as a result of the intermolecular interaction.

SEM pictures revealed clear paralelipipedic crystals for genistein while an irregular shape can be noticed for its ester with the fatty acid. Following complexation with BCD, which exhibits an amorphous structure, an amorphous product was obtained indicating the formation of a distinct new product as a result of the intermolecular interaction (Figure 2).

**Figure 3:**
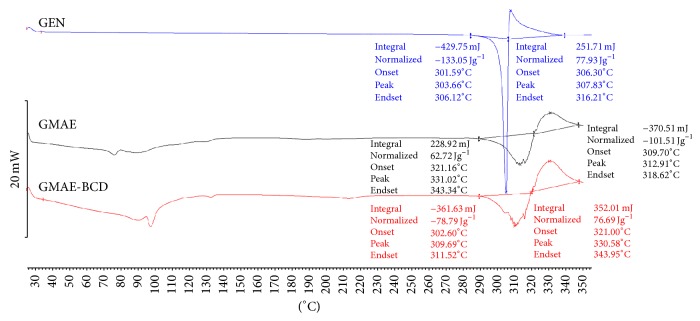
DSC pictures for (a) GEN, sharp endothermic peak around 303°C abruptly followed by a much smaller exothermic one at 307°C, indicating a melting of the active compound probably accompanied by a chemical decomposition, (b) GMAE, one endothermic and one exothermic peak, slightly shifted towards higher temperatures, 313°C and 331°C, respectively, and (c) GMAE-BCD, similar profile with GMAE. A small water loss can be noticed for both GMAE and GMAE-BCD at around 100°C, indicating the loss of the “internal” water molecules of the cyclodextrin which were replaced by the guest molecule.

The DSC curve of genistein displays a sharp endothermic peak around 303°C abruptly followed by a much smaller exothermic one at 307°C, indicating a melting of the active compound probably accompanied by a chemical decomposition. BCD (not shown) displays an almost flat curve, the melting process starting around 350°C. Genistein's ester with the myristic acid exhibits a similar thermal profile, with an endothermic and an exothermic peak, slightly shifted towards higher temperatures, 313°C and 331°C, respectively. These peaks can also be noticed in the DSC curve recorded for the inclusion complex GMAE-BCD. The preservation of genistein's characteristic peaks in the thermal analysis of its ester inclusion complex suggests that only the myristoyl moiety was included inside the CD cavity, the bulk of GEN molecule being left outside the complex. A small water loss can be noticed for both GEN ester and its cyclodextrin binary product at around 100°C, indicating the loss of the “internal” water molecules of the cyclodextrin which were replaced by the guest molecule. The DSC curves are exhibited in Figure 3.

**Figure 4:**
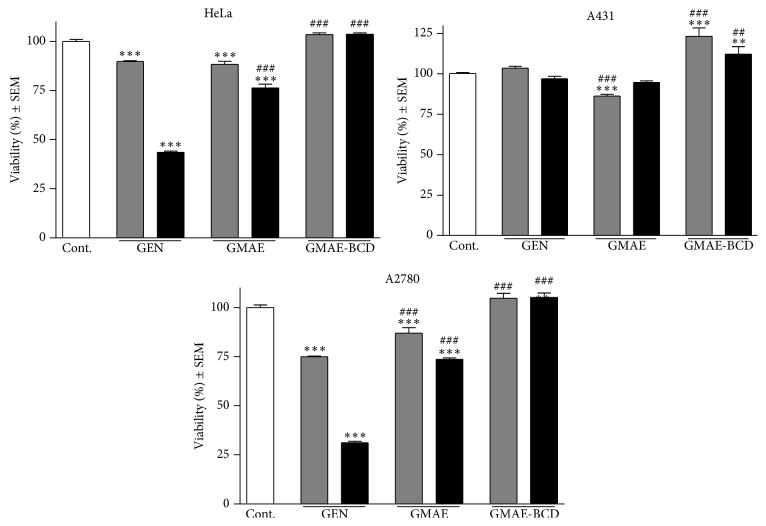
Viability of selected cells after incubation with 10 *μ*M (gray) or 30 *μ*M (black) of GEN, GMAE, and GMAE-BCD. *∗∗* and *∗∗∗* indicate *p* < 0.01 and *p* < 0.001, respectively, as compared with the control cells. ## and ### indicate *p* < 0.01 and *p* < 0.001, respectively, as compared with the cells treated with the corresponding concentration of GEN.

The biological activity of tested compounds on the HeLa (cervix adenocarcinoma), A2780 (ovary carcinoma), and A431 (skin epidermoid carcinoma) cell lines is depicted in Figure 4.

MTT proliferation assay on HeLa (cervix adenocarcinoma) cell line shows that after a 72 h incubation period using 10 *μ*M genistein in DMSO the cell viability reaches 89,70%; at the same concentration, GEN esterification has practically no effect on cell viability, the percentage of viable cells reaching 88,21% after incubation with GMAE. The application of beta-cyclodextrin-encapsulated GMAE led to 103,41% of viable cells. However, using the same cell line and a concentration of 30 *μ*M tested compounds, the percentage of viable cells decreased to 43,54% for GEN. At the same concentration, GMAE managed to reduce cell viability to 76,23% while after entrapment inside BCD it produced 103,52% of viable cells.

In case A431 (skin epidermoid carcinoma) cell line was used, at the two previously mentioned concentrations (10 *μ*M and 30 *μ*M) of tested compounds and following a 72 h incubation period, GEN and its products exhibited weaker antiproliferative activity compared to HeLa cells. Thus, for 10 *μ*M GEN, 103,61% cell viability was recorded; GMAE revealed a slightly higher activity, reaching a percentage of 86,34% viable cells. On the contrary, 123,24% viable cells were registered for GMAE-BCD. The concentration of 30 *μ*M failed to produce significant changes: 97,03%, 94,73%, and 112,33% viable cells were reported for GEN, GMAE, and GMAE-BCD, respectively.

In case of the last tested cell line, A2780 (ovary carcinoma), using the same concentrations (10 *μ*M and 30 *μ*M) and incubation time (72 h), the application of 10 *μ*M GEN or its products, respectively, resulted in the following viability percentages: 74,80% for GEN, 86,95% for GMAE, and 104,72% for GMAE-BCD. The concentration of 30 *μ*M tested compounds considerably reduced cell viability as follows: 31,13% and 73,60% for GEN and GMAE, respectively; however, 105,023% viable cells were determined as a result of GMAE-BCD incubation.

The novelty brought by this study resides in the esterification of genistein with fatty acids followed by cyclodextrin encapsulation. Both chemical and enzymatic esterification of flavonoids are useful approaches to increase cellular permeability and, by consequence, biological activity [25]. Mellou et al. showed that acylation with polyunsaturated fatty acids can lead to increased antitumor and antiangiogenic properties [26]. They have concluded that the preparation of lipophilic flavonoid esters via derivatization of their hydroxyl groups may generate an increased stability in lipophilic media [27]. Also, it was reported that esterification with short side-chain fatty acids may enhance penetration through phospholipid membranes [28].

One of the goals of the present study was to synthesize a new ester of genistein, using the myristic acid as reaction partner. The formation of ester derivative was confirmed by means of IR and mass spectroscopy. In the second phase of the study, the ester was submitted to cyclodextrin complexation in order to ensure higher water solubility and, therefore, increased concentration in the biological medium. The complexes were evaluated using spectroscopic and thermal analysis as well as scanning electron microscopy, methods that revealed the existence of true intermolecular inclusion. Given the ester structure and also the experimental findings, one can assume that the myristoyl moiety was included inside the cyclodextrin cavity while the genistein molecule lies outside the toroidal ring. This molecular orientation provides the necessary increase in water solubility, due to the presence of the hydrophilic cyclodextrin, but also preserves the properties of genistein, both physicochemical and biological properties. Nevertheless we were able to accomplish a highly soluble water product that is still active on the target sites due to the genistein moiety which is not embedded inside cyclodextrin cavity. Moreover, in case of* in vivo* experiments, after the cleavage of the inclusion complex the free ester may exert its biological activity as such or following decomposition under esterase activity.

The second goal of this study was to investigate the antiproliferative activity of the newly synthesized GEN derivatives on selected cancer cell lines: HeLa, A431, and A2780. GEN was previously reported as an active agent on HeLa cells; a recent study showed that the antiproliferative mechanism involves the modulation of matrix metalloproteinase-9, the tissue inhibition of matrix metalloproteinase-1, and the inhibition of topoisomerase II *α* expression [29]. Furthermore, GEN was reported to induce apoptosis in HeLa cells via caspase activation [30]; also, using a concentration similar to that used in the current study, namely, 25 *μ*M, GEN was able to sensitize HeLa cells to cisplatin [31].

Literature reports that GEN was found to prevent UV radiation-induced apoptotic biochemical changes in A431 cells [32]; also, the incubation of A431 cells with GEN leads to decreased EGF-stimulated serine, threonine, and tyrosine phosphorylation [33]. However, most reported studies confirm the experimental results of the present paper that revealed a poor antiproliferative activity of GEN or its derivatives on A431 cell line.

Human ovarian cancer cells A2780 were sensitized to cisplatin after exposure to GEN [34]. In a recent study the isoflavone was reported as highly active agent against several human ovarian cancer cells, exhibiting antiproliferative, proapoptotic, antioxidant, and antiangiogenic properties [35]. Apoptosis and autophagocytosis following GEN incubation were also depicted by Gossner et al. [36]. The experimental results of the current study are consistent with previously reported data, the 72 h exposure to 30 *μ*M GEN leading to the decrease of A431 cells' viability to 31,13%.

Altogether, we noticed a poor antiproliferative activity of GEN when applied on A431 cancer cell line while the other two tested cell lines, HeLa and A2780, proved sensitive to the active drug; also, the higher concentration used in the study, 30 *μ*M, led to higher antiproliferative activity on both tumor cell lines. The esterification of GEN with the fatty acid has resulted in weaker* in vitro* antiproliferative activity on all three cell lines; this behavior can be explained by the highly increased lipophilicity of the ester molecule, which cannot be easily solubilized in aqueous environment; therefore, the active concentration accomplished in the biological medium is not sufficient to provide significant antitumor effect. The inclusion of the fatty acid moiety inside the water soluble cyclodextrin highly improves the product's aqueous solubility; however, as previously reported in the literature [37], fatty acids exhibit a very strong affinity for cyclodextrin complexation, leading to highly stable complexes. Therefore, we can assume that the stability of the resulting complex between BCD and GMAE is so high that the biological* in vitro* environment does not provide the necessary physicochemical and enzymatic tools to release the guest molecule (GMAE) and to destroy the ester link in order to generate the active genistein. Some previous studies [23, 38, 39], focused on betulin and betulinic acid, have revealed similar behavior; thus, after the inclusion of the active drug inside a highly hydrophilic cyclodextrin, with the formation of very stable complexes, the* in vitro* tests led to rather poor antiproliferative results. However,* in vivo* tests on animal models showed strong antitumor properties in terms of tumor development and metastasis. This anticancer activity is presumably due to the bioactivation of the complex in the biologic environment, followed by the release of the active drug previously entrapped inside the cyclodextrin's cavity.

All these considered, we assume that during future* in vivo* experiments, the BCD complex of GMAE will be destroyed in the living tissues and, under the influence of various esterases, the released active drug, GMAE, will be cleaved to the corresponding fragments, active GEN, and myristic acid. In this presumable scenario, the cyclodextrin complex provides the necessary water solubility to accomplish effective concentrations in the biological environment while the increased lipophilicity of GMAE offers an improved membrane passage inside the cancer cells.

## 4. Conclusion

The present study focuses on the synthesis and analysis of a GEN ester derivative with myristic acid followed by BCD encapsulation; physicochemical analysis using consecrated techniques such as FTIR, MS, DSC, and SEM revealed both a successful esterification and inclusion inside the cyclodextrin cavity. The* in vitro* biological analysis exhibited rather poor antiproliferative results on all three tested cancer cell lines, behavior that may be due to the high stability of the complex within the* in vitro* environment; future* in vivo* studies are needed in order to fully assess the potential of such derivatives as anticancer agents.
